# Supplementary material for: Bugs on Drugs: Paracetamol Exposure Reveals Genotype-Specific Generational Effects on Life History Traits in Drosophila melanogaster
Source: Insects. 2024 Oct 1;15(10):763. doi: 10.3390/insects15100763 (PMC11509061; doi:10.3390/insects15100763)
Supplement: Supplementary file 1 [file insects-15-00763-s001.zip › Supplement_Article_Paracetamol_Clean_Proof.pdf]

Supplemental material for:

Bugs on drugs: Paracetamol exposure reveals genotype specific generational effects on life history traits in *Drosophila melanogaster*

Birk Nete Randlev Gleerup Hundebøl[1], Palle Duun Rohde[2], Torsten Nygaard Kristensen[3], Rune Wittendorff Mønster Jensen[4,5], Thomas Vosegaard[4,5], Jesper Givskov Sørensen[1#]

[1] Department of Biology, Aarhus University, Aarhus, Denmark

[2] Department of Health Science and Technology, Aalborg University, Aalborg, Denmark

[3] Department of Chemistry and Bioscience, Aalborg University, Aalborg, Denmark

[4] Interdisciplinary Nanoscience Center (iNANO), Aarhus University, Aarhus C, Denmark

[5] Department of Chemistry, Aarhus University, Aarhus C, Denmark

[#] Corresponding author: Jesper Givskov Sørensen, Department of Biology, Ny Munkegade 114-116, 8000 Aarhus C, Denmark, e-mail: jesper.soerensen@bio.au.dk

Detailed Materials and Methods

NMR experiment and paracetamol concentration

As samples were prepared by taking a known amount (m_{DSS}) of DSS and dissolve it in $V_{tot} =$ 40 mL H_2O, the concentration of DSS is

$$C_{DSS} = \frac{m_{DSS}}{M_{DSS}V_{tot}}$$

The NMR samples were prepared by weighing out some mg of food sample (m_{food}) and suspend it in 900 µL of the DSS solution and add deuterated water to a total volume of $V_{sample} = 1$ mL. See the actual amounts of food and DSS in Table S7 below. The final DSS concentration then becomes

$$C_{DSS} = 0.9\frac{m_{DSS}}{M_{DSS}V_{tot}}$$

With NMR, we may calculate the relative concentrations of CSS and paracetamol. We find

$$C_{Para} = C_{DSS}X$$

X comes from the measured intensities, but they should be normalized to the number of hydrogens for the specific signal. We have used the signal at 0.0 ppm for DSS, which represents the three methyl groups of DSS, hence corresponding to 9 hydrogens. For paracetamol, we use the methyl group signal at 2.14 ppm, hence corresponding to 3 hydrogens. These two signals were chosen for the measurement as they did not overlap with other signals. The integrals were done in intervals of 0.000 ± 0.025 ppm and 2.140 ± 0.025 ppm, respectively. Knowing the integrals, X is given by

$$X = 3\frac{I_{2.14}}{I_{0.0}}$$

Table S8 lists the intensity ratio $I_{2.14}/I_{0.0}$ for all samples. From these values we calculate paracetamol concentration relative to the food mass and normalize to the Day 0 average. This data is listed in Table S9 and plotted in Figure S1.

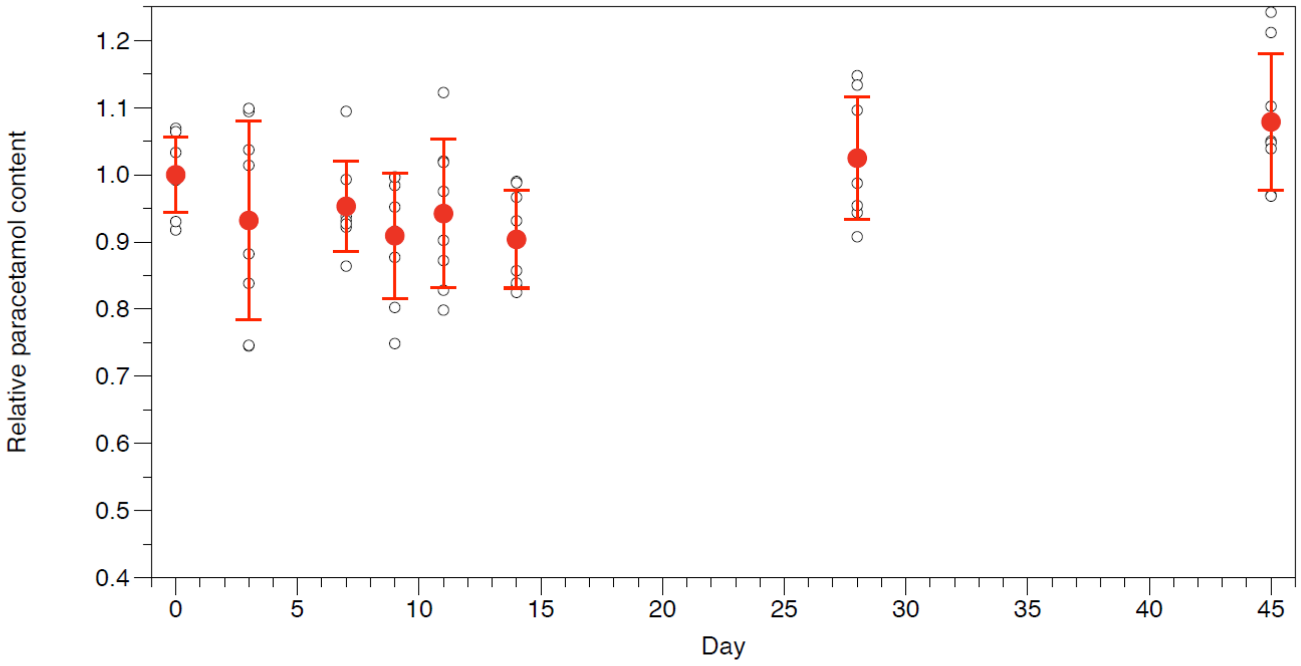

Figure S1. Measured concentration of paracetamol in 7 mL vials of Carolina instant fly food spiked with paracetamol at different days after creation as determined by NMR.

Table S1 Overview of exact amount (mg) paracetamol added to the 7 mL food vials for the dose-response curve of the outbred and the isofemale line (above) and the seven isofemale line for the phenotypic investigations (below). Target refers to the calculated amount for each concentrationValues are based on the actual weighted amount of paracetamol.

**Experiment I**			
**Concentration (mM)**	**Outbred**	**Isofemale**	**Target**
10	10.578	10.691	10.581
20	21.156	21.255	21.162
30	31.733	31.691	31.744
40	42.389	42.382	42.325
50	52.967	52.945	52.906
60	63.622	63.509	63.487
70	73.889	73.818	74.068
80	84.544	84.636	84.650
90	95.200	95.200	95.231
100	106.011	106.018	105.812
**Experiment II**			
**Concentration (mM)**	**L1-L3**	**L4-L7**	**Target**
20	21.14	21.126	21.162
40	42.336	42.35	42.325

Table S2. Sample size (flies assayed) for each trait, generation, dose and line.

Line / Trait	Gen\Dose	**L1**			**L2**			**L3**			**L4**			**L5**			**L6**			**L7**		
		0	20	40	0	20	40	0	20	40	0	20	40	0	20	40	0	20	40	0	20	40
**Fecundity**	F0	55	44	40	40	30	53	56	54	55	40	40	39	40	39	43	40	39	34	40	40	43
	F1	38	39	38	36	33	38	40	40	38	40	21	40	40	30	39	40	24	40	40	40	12
	F2	40	38	39	34	40	39	40	40	40	40	39	38	40	40	39	40	40	39	38	40	8
**Longevity**	F0	38	53	42	49	39	54	36	57	44	46	62	45	68	67	59	52	61	69	51	53	60
	F1	54	67	-	23	15	49	57	63	64	54	61	59	47	65	72	75	57	69	44	89	62
	F2	70	58	79	22	45	66	64	72	73	68	69	65	49	69	64	52	49	54	52	67	15
**Activity (young)**	F0	47	66	61	41	46	71	62	70	72	67	65	53	54	63	66	65	66	70	67	65	65
	F1	68	72	72	39	55	60	68	72	69	66	67	66	66	66	66	66	66	66	66	66	66
	F2	65	62	60	28	71	59	58	59	62	66	66	71	64	66	60	60	64	69	63	66	8
**Activity (old)**	F0	44	62	-	47	38	-	43	63	-	42	55	35	58	67	72	58	67	72	53	60	64
	F1	52	54	56	15	17	5	68	53	54	38	37	33	68	63	56	68	63	56	38	60	57
	F2	46	49	60	1	22	11	53	48	53	2	12	52	48	60	29	48	60	29	44	43	8

Table S3. Statistical overview of the fecundity model using lines as a random factor.

**Fecundity**	**SqrSumOffspring**		
Predictors	Estimates	CI	p
(Intercept)	2.54	2.07 – 3.01	<**0.001**
Dose [D20]	1.04	0.74 – 1.34	<**0.001**
Dose [D40]	0.94	0.65 – 1.23	<**0.001**
Gen [F1]	0.53	0.23 – 0.83	**0.001**
Gen [F2]	1.51	1.21 – 1.81	<**0.001**
Dose [D20] × Gen [F1]	-0.84	-1.28 – -0.39	<**0.001**
Dose [D40] × Gen [F1]	-0.76	-1.19 – -0.33	**0.001**
Dose [D20] × Gen [F2]	-1.57	-2.00 – -1.14	<**0.001**
Dose [D40] × Gen [F2]	-1.58	-2.01 – -1.14	<**0.001**
Random Effects			
σ^2	3.41		
$\tau_{00\ Line}$	0.33		
ICC	0.09		
N_{Line}	7		
Observations	2445		
Marginal R^2 / Conditional R^2	0.041 / 0.126		

Table S4. Statistical overview of the longevity model using lines as a random factor.

**Longevity**	**SqrDays**		
Predictors	Estimates	CI	p
(Intercept)	5.81	5.41 – 6.21	**<0.001**
Dose [D20]	-0.13	-0.26 – -0.00	**0.048**
Dose [D40]	-0.04	-0.17 – 0.09	0.572
Gen [F1]	-0.23	-0.36 – -0.09	**0.001**
Gen [F2]	-0.32	-0.45 – -0.19	**<0.001**
Dose [D20] × Gen [F1]	0.31	0.13 – 0.49	**0.001**
Dose [D40] × Gen [F1]	0.13	-0.06 – 0.31	0.172
Dose [D20] × Gen [F2]	0.12	-0.06 – 0.30	0.177
Dose [D40] × Gen [F2]	0.00	-0.18 – 0.19	0.960
Random Effects			
σ^2	0.80		
$\tau_{00\ Line}$	0.27		
ICC	0.26		
N_{Line}	7		
Observations	3473		
Marginal R^2 / Conditional R^2	0.015 / 0.266		

Table S5. Statistical overview of the spontaneous locomotor activity model using lines as a random factor.

**Spontaneous Locomotor Activity**	**LogDistance**		
Predictors	Estimates	CI	p
(Intercept)	0.44	0.37 – 0.52	**<0.001**
Dose [D20]	0.02	-0.02 – 0.05	0.369
Dose [D40]	-0.02	-0.06 – 0.02	0.361
Gen [F1]	-0.03	-0.07 – 0.01	0.145
Gen [F2]	-0.17	-0.21 – -0.13	**<0.001**
Age [Young]	0.24	0.20 – 0.27	**<0.001**
Dose [D20] × Gen [F1]	-0.05	-0.10 – 0.01	0.089
Dose [D20] × Gen [F2]	0.11	0.06 – 0.17	**<0.001**
Dose [D40] × Gen [F1]	-0.05	-0.11 – 0.01	0.079
Dose [D40] × Gen [F2]	0.05	-0.01 – 0.11	0.106
Dose [D20] × Age [Young]	-0.00	-0.05 – 0.05	0.956
Dose [D40] × Age [Young]	-0.01	-0.07 – 0.04	0.588
Gen [F1] × Age [Young]	0.13	0.09 – 0.18	**<0.001**
Gen [F2] × Age [Young]	0.15	0.10 – 0.19	**<0.001**
Dose [D20] × Gen [F1] × Age [Young]	-0.00	-0.07 – 0.06	0.899
Dose [D20] × Gen [F2] × Age [Young]	-0.18	-0.25 – -0.11	**<0.001**
Dose [D40] × Gen [F1] × Age [Young]	-0.00	-0.08 – 0.07	0.901
Dose [D40] × Gen [F2] × Age [Young]	0.03	-0.04 – 0.11	0.375
Random Effects			
σ2	0.02		
$\tau_{00\ ID}$	0.06		
$\tau_{00\ Line}$	0.01		
ICC	0.81		
N_{ID}	6626		
N_{Line}	7		
Observations	171628		
Marginal R^2 / Conditional R^2	0.218 / 0.853		

Table S6. Overview of the post hoc Dunn's test, comparing individual groups around the drop of the dose-response curve with the control group.

Group 1	Group 2	n_1	n_2	Statistic	p	p adjusted
D0	D30	15	15	1.294244	0.196	0.919
D0	D40	15	15	0.36029	0.719	0.926
D0	D50	15	15	-1.32922	0.184	0.919
D0	D60	15	15	-3.60989	0.000	**0.003**
D0	D70	15	15	-4.6278	0.000	**0.000**

Table S7. Amounts of DSS and paracetamol for the different NMR samples.

Day		0	3	7	9	11	14	28	45
m DSS (mg)	Replicate	13.7	10.1	12.4	12.2	10.6	11.5	12.0	11.3
m paracetamol (mg)	1	70.1	55.9	88.4	68.7	53.3	87.1	84.6	80.3
	2	59.2	54.1	58.5	62.1	56.1	61.9	61.1	89.7
	3	69.4	80.7	69.1	86.6	61.7	64.8	88.1	87.1
	4	64.2	95.1	91.9	53.5	71.5	63.5	63.5	65.6
	5	56.0	95.2	85.7	79.3	76.2	71.9	90.3	90.8
	6	58.4	78.6	69.6	61.1	74.1	53.0	84.2	85.0
	7	81.8	76.3	62.6	70.5	72.5	67.5	63.7	86.6
	8	63.9	106.2	76.5	56.4	79.0	63.7	91.1	66.8

Table S8. The intensity ratio between the paracetamol and DSS peaks [$I_{2.14}/I_{0.0}$].

Day/Replicate	0	3	7	9	11	14	28	45
1	0.625	0.752	0.887	0.646	0.611	0.787	0.830	0.935
2	0.486	0.737	0.547	0.634	0.598	0.655	0.726	1.055
3	0.669	1.008	0.763	0.777	0.582	0.578	0.902	1.171
4	0.582	1.218	0.869	0.412	0.694	0.589	0.722	0.805
5	0.536	1.030	0.801	0.791	0.910	0.653	1.063	1.240
6	0.496	0.811	0.653	0.625	0.764	0.571	0.838	0.918
7	0.737	0.709	0.606	0.689	0.961	0.725	0.605	0.934
8	0.607	0.998	0.671	0.468	0.950	0.644	0.973	0.773
Average	0.592	0.908	0.725	0.630	0.759	0.650	0.832	0.979

Table S9. Relative concentrations of paracetamol in the various samples normalized to the Day 0 average.

Day/Replicate	0	3	7	9	11	14	28	45
1	0.986	1.096	1.004	0.925	0.980	0.838	0.950	1.061
2	0.907	1.110	0.936	1.004	0.911	0.981	1.149	1.072
3	1.065	1.017	1.104	0.883	0.806	0.827	0.991	1.225
4	1.002	1.043	0.946	0.757	0.830	0.860	1.100	1.118
5	1.057	0.882	0.934	0.982	1.021	0.842	1.140	1.244
6	0.938	0.841	0.938	1.006	0.881	0.998	0.963	0.984
7	0.996	0.756	0.968	0.962	1.133	0.997	0.919	0.983
8	1.050	0.765	0.877	0.817	1.028	0.938	1.034	1.055
Average	1.00±0.06	0.94±0.15	0.96±0.07	0.92±0.09	0.95±0.11	0.91±0.08	1.03±0.09	1.09±0.10
